# Exploring the capacity of the Somaliland healthcare system to manage female genital mutilation / cutting-related complications and prevent the medicalization of the practice: a cross-sectional study

**DOI:** 10.1186/s12913-020-5049-2

**Published:** 2020-03-12

**Authors:** Mohamed Yussuf, Dennis J. Matanda, Richard A. Powell

**Affiliations:** 1 Population Council, Avenue 5, Rose Avenue, P.O. Box 17643-00500, Nairobi, Kenya; 2 MWAPO Health Development Group, P.O. Box 459-00621, Village Market, Nairobi, Kenya

**Keywords:** Female genital mutilation/cutting, FGM/C, Healthcare system, Somaliland, Somalia, Africa

## Abstract

**Background:**

Female genital mutilation/cutting (FGM/C) negatively impacts the wellbeing of girls and women throughout their lifecycle. In Somalia, FGM/C prevalence is nearly universal (98%) among females aged 15–49 years, with infibulation prevalence at 77%. Whilst there is need to engage healthcare workers in the prevention and management of FGM/C, minimal information exists indicating healthcare systems’ capacity to fulfil this role. This study explored factors impacting the capacity of the Somaliland healthcare system to prevent the medicalization, and manage the complications of, FGM/C.

**Methods:**

A cross-sectional qualitative study using semi-structured key informant interviews, conducted in the Somali language, was undertaken in the Maroodi Jeex and Awdal regions of Somaliland, in rural and urban Borama and Hargeisa districts in December 2016. A total of 20 interviews were conducted with healthcare workers comprised of medical doctors, nurses, midwives and system administrators. Transcribed and translated interview data were analysed using the template analysis approach.

**Results:**

Healthcare workers reported understanding the adverse impact of FGM/C on the health of girls and women. However, they faced multiple contextual challenges in their preventative and management roles at the *individual level*, e.g., they lacked specific formal training on the prevention and management of FGM/C complications and its medicalization; *institutional level*, e.g., many facilities lacked funding and equipment for effective FGM/C management; and *policy level*, e.g., no national policies exist on the management of FGM/C complications and against its medicalization.

**Conclusion:**

Healthcare systems in urban and rural Somaliland have limited capacity to prevent, diagnose and manage FGM/C. There is a need to strengthen healthcare workers’ skill deficits through training and address gaps in the health system by incorporating the care of girls and women with FGM-related complications into primary healthcare services through multi-sectoral collaboration and coordination, establishing clinical guidelines for FGM/C management, providing related equipment, and enacting policies to prevent the medicalization of the practice.

## Background

Female genital mutilation / cutting (FGM/C) entails all procedures involving the partial or total removal of the external female genitalia or other injury to the female genital organs for non-medical reasons [1]. There are four main types of FGM/C, the most severe being Type III: infibulation, also referred to as pharaonic [2]. Globally, an estimated 200 million girls and women have undergone the cut [3], and approximately 70 million girls aged 0–14 years are at risk of being cut [4].

Historically performed by elderly women, barbers or traditional birth attendants, FGM/C is a physically invasive procedure often associated with multiple adverse impacts [5–7]. Immediate potential complications include, among others, infection or abscess formation, septicemia, shock, and death [8–10]. Longer-term complications can include pain, scarring, urinary tract infections [11], and poor obstetric and neonatal outcomes [12], among others [10, 13, 14].

In Somalia, FGM/C prevalence is nearly universal (98%) among females aged 15–49 years, with infibulation prevalence at 77% among the same group [15]. In 2011 in the self-declared state of Somaliland, prevalence was 99 and 85%, respectively [16]. In a 2016 study among 25 communities living in the Maroodi Jeex and Togdheer regions reported prevalence at 99%, with 80% having undergone infibulation [17]. Despite its high prevalence, differences exist in support for the continuation of FGM/C. In Somaliland, only 29% of females aged 15–49 years favoured its continuation in 2011, compared with 65% in Somalia in 2006 [16].

Deeply rooted in custom, the social determinants underpinning FGM/C are many and diverse including, among others, rites of passage and cultural obligation, the preservation of chastity, and enhancing female marriageability prospects [18–26]. These factors often operate dynamically at the individual, family, community and national levels [27]. In Somaliland, the popularity of the practice is strongly embedded in the Somali culture, despite the decades-long global campaign to eliminate it [28]. The majority of community members (84%) living in Somaliland intend to cut their daughters in the future, with women in particular intending to select a less severe cut that they perceive the community expects them to use [17].

Programmatic work to prevent and eliminate FGM/C has been initiated that supports the United Nations’ Sustainable Development Goals [29] by strengthening the World Health Organization’s (WHO) six health system framework building blocks, which includes the health workforce [30]. In 2016 the WHO disseminated guidelines for healthcare workers, managers and policymakers on caring for FGM/C-affected girls and women, service planning, and developing and implementing national and local healthcare protocols and policies [31]. However, while such efforts to address the practice have focused on prevention, less attention has been devoted to treating health complications, building the capacity of healthcare workers to provide optimal care, and engaging care providers as potential agents of behavioural change [32, 33].

In high-income countries, especially those with immigrant communities from high FGM/C-prevalent countries, health systems’ challenges in addressing the needs of those affected are widely reported [34]. Examples include limited staff knowledge around the concept, typology, and prevalence of FGM/C and the protocols for remedial action [33–41]. Similar FGM/C healthcare capacity limitations have been found in low-income settings. In sub-Saharan Africa (SSA), one of the few studies investigating healthcare workers’ capacity to manage FGM/C-related complications revealed challenges regarding knowledge and skill sets [42].

Healthcare providers are potentially critical actors in the prevention of FGM/C and care for its survivors. However, in SSA a considerable number of healthcare workers are either engaging in the practice as cutters, or supporting its continuation. A cross-sectional study of 1288 healthcare providers in six countries found that 25% embraced its continuation, and 24% expressed their intention of subjecting their own daughters to FGM/C [42]. Moreover, the willingness to continue the practice increased among healthcare providers operating in rural-based health facilities, with 43% embracing its continuation and nearly half (47%) intending to subject their own daughters to the practice [43]. Healthcare in Somaliland, as with other Somalia ‘zones’, is largely in the private sector, regulated by the Ministry of Health of the Federal Government of Somalia. The system is largely staffed by undertrained, under-supervised and -paid staff, dependent upon donations from international agencies. The national health system is implemented through the Essential Package of Health Services designed in 2008 to provide a common framework and minimum standards for the delivery of health services in within the framework of the health sector strategic plans. The essential package is implemented across four levels of service provision, each with a standardized service profile and each supported by a standardized set of management and support components and helps define health systems standards for the government, the United Nations, non-governmental agencies and private service providers [44].

In Somaliland, evidence exists of healthcare workers involvement in cutting, although most report intending to decline requests to cut a girl [17], a trend towards the medicalization of FGM/C that has been noted in other African countries [42, 43, 45].

Despite high FGM/C prevalence and an increase in the practice’s medicalization in Somaliland, minimal evidence exists on the capacity of healthcare providers and the health system to prevent medicalization of FGM/C and manage FGM/C-related complications. This study therefore investigated the capacity of the healthcare system to manage FGM/C cases and prevent the medicalization of the practice by exploring healthcare workers’ knowledge, training and skills.

## Methods

### Design and setting of the study

A cross-sectional qualitative study utilizing semi-structured, key informant interviews was undertaken in the Maroodi Jeex (also known as Waqooyi Galbeed) and Awdal regions of Somaliland, in rural and urban Borama and Hargeisa districts in December 2016. Maroodi Jeex is an administrative region and the most populous region in Somaliland, with Hargeisa its capital city. With a population of 1,242,003 [46], it is mainly inhabited by people from the Somali ethnic group of the Isaaq clan [47] and is a very strategic region, with rich farmlands and large ports [48]. The Awdal region is the most westerly province of Somaliland. It borders Maroodi Jeex to the East, Djibouti to its North-West, Ethiopia to its South and South-West lies, and the Gulf of Aden to its North, with a population of 673,264 [46]. The regional capital is Borama, the largest city of the North-Western Awdal region, which is the commercial seat of the province, situated near the Ethiopian border.

Healthcare is provided across four organisational levels in Somaliland: primary health unit, health centre, referral health centre and hospital. Interviewees were selected from primary health units, health centres and hospitals, both public and private, located in the two districts. The health professionals were at least 20 years of age, and able to at least speak—and ideally read—the local language in which the interview was conducted. All respondents worked in the study communities for at least 2 years, addressed FGM/C in the course of their work (i.e., managing FGM/C-related complications and preventing the medicalization of the practice) and belonged to one of the following cadre: doctors, nurses, midwives, clinical managers and health system administrators. All interviews were held at a location that was acceptable to the selected study participants.

### Data collection tools

The interview guide (see Additional file 1) was developed by the research team based on their knowledge of the study sites and the existing FGM/C literature. The tools were then reviewed by FGM/C research experts and in-country Ministry of Labour and Social Affairs officials, with changes made to enhance clarity and understanding. The translated tools were pilot tested with a small number (*n* = 5) of health workers—one from each of the five cadre categories—to ensure they were comprehendible, and culturally appropriate and valid. Findings from the piloting phase were not included in the final data set for analysis. The tools’ content included questions on healthcare providers’ medical knowledge of FGM/C complications, their clinical skills and formal training to manage clients with such complications, the perceived capacity of the healthcare system to prevent girls and women from undergoing FGM/C, and the availability of national FGM/C policies, including against its medicalization.

Experienced research assistants were trained on conducting research in an ethically appropriate manner and qualitative data collection approaches during a one-week workshop. Key informant interviews were conducted in the local Somali language and digitally audio recorded. Data on the socio-demographic characteristics of study participants were also collected using a bespoke basic tool. Recorded interviews underwent a rigorous multi-stage, forward-and backward transcription-translation process with bi-lingual staff to ensure data quality. Differences detected between the Somali and English language versions were identified and discussed between the transcribers and the translators until agreement was reached.

### Data management and analysis

Finalized translated transcripts were thematically analysed using the template analysis approach with NVivo v11® software [49]. The template analysis approach is a pragmatic, iterative approach combining deductive and inductive analyses, producing a non-hierarchical coding framework, or “template” [50]. The template was developed from a priori themes and sub-themes covered in the study’s data collection tool. Each code in the framework was described with specific definitions and reviewed for internal consistency; this was especially important as the analysts often worked in different locations.

The developed template was applied to a sample of interview transcripts (i.e., two key informant interviews) to ensure it covered most anticipated thematic codes and sub-codes. It was then applied to the remaining transcripts, with template revisions made for any additional emerging themes. To ensure methodological rigor, in addition to using researcher triangulation, nter-rater coding reliability was assessed by running a coding comparison query among a random sample of three coded transcripts. This produced a Kappa score of 0.81 score, with 0.75 indicating the minimum threshold for excellent agreement [51]. The study engaged two key stakeholders in Somaliland with extensive experience working in FGM/C as participant proxies with data checking to ensure researchers’ interpretations had veracity.

### Ethical approvals

Written informed consent was obtained from all participants. The study received ethical approval from the Population Council’s Institutional Review Board (Ref: Protocol 775) and Somaliland’s Ministry of Labour and Social Affairs (Ref: W/SH/AB/02/28/016).

## Results

### Characteristics of participants

Table 1 summarises the participants’ socio-demographic characteristics. The study population was comprised of 20 healthcare professionals: four medical doctors, four clinical managers, four nurses, four midwives and four system administrators. For inclusion in the study they had to be at least 20 years of age and worked at a health facility in a rural or urban area for at least 1 year. The majority of participants were female (*n* = 12), had an age range between 26 and 52 years and all had secondary education and above. All participants identified as Sunni Muslims, while 17 were married.

**Table 1:** Participants’ socio-demographic characteristics

Characteristics	*N*	%
**Sex**		
Male	8	40
Female	12	60
**Age**		
20–29 years	6	30
30–39 years	6	30
40–49 years	5	25
50–59 years	3	15
**Education level**		
Above secondary	20	100
**Marital status**		
Married	17	85
Unmarried	3	15
**Religion**		
Sunni Muslim	20	100
**Profession**		
System administrators	4	20
Clinical manager	4	20
Medical doctor	4	20
Nurse	4	20
Midwife	4	20
**Total**	**20**	**100**

In analysing factors impacting the capacity of the Somaliland healthcare system to prevent the medicalization of FGM/C and manage FGM/C-linked complications, study findings are presented based on thematic areas of interest beginning with factors at the individual, institutional and, lastly, policy levels.

#### Individual level

At this level, we explored the knowledge of healthcare workers to diagnose and treat FGM/C complications; availability of health workers with skills to conduct de-infibulations and clitoral reconstruction; prevention of FGM/C; healthcare providers’ support for the Sunna cut, a supposedly less severe form of FGM/C which, given varying understanding of its precise meaning, is not easily equated using the official WHO FGM/C classifications; and medicalization of FGM/C by health professionals.

##### Ability of healthcare providers to diagnose and treat FGM/C complications

Respondents were aware of some of the harmful physical, psychological and social consequences of FGM/C. However, except for a few specialist doctors and gynaecologists, most had not received pre- or in-service training to identify and manage FGM/C-related health complications. They therefore reported a need for specialised training on FGM/C management as most primarily relied on their general knowledge to address such complications. As two doctors reported:

“I didn’t get training to handle a cut girl. I would like to recommend it for myself to get training in order to handle girls who have these complications resulting from FGM/C.” Medical Doctor, Borama

“I think the skills and training to manage complications of FGM/C is very low. Most … people are nurses and midwives who are locally trained and have no specialized training on this. In our country, there are few specialist doctors … who are trained for such tasks. So, healthcare workers who are doctors … should be trained adequately to manage FGM/C complications of those who were cut.” Medical Doctor, Borama

##### Availability of healthcare workers with skills to conduct deinfibulation and clitoral reconstruction

Most providers felt that management of FGM/C complications were problematic given they lacked the necessary skills, especially for de-infibulation and clitoral reconstruction which, whilst not in major demand as a surgical procedure, was considered an important skill to possess and required them to refer elsewhere. Additionally, very few were trained to conduct episiotomies during labour for those with FGM/C and to offer psychosocial support.

“Healthcare workers with enough skills to manage FGM/C are not many and they are not mostly in the places where the practice is done.” Nurse, Hargeisa

“There are those women who have many health complications. They come saying, ‘I want to be deinfibulated’. So, we refer them to gynaecologists. It is done in big hospitals. We refer because our staff do not have the necessary skills.” Midwife, Borama

##### Healthcare workers’ attitudes towards FGM/C

Most healthcare workers supported the prevention and discontinuation of FGM/C, especially infibulation, because of the detrimental effects on the physical, social and psychological wellbeing of girls and women. However, a few healthcare workers supported the continuation of the less severe type of FGM/C—the Sunna type—arguing that this type of FGM/C is recommended by Islam and poses no health risks to girls and women. As two doctors noted:

“It is better not to do it because it causes health complications … I say, since it has very negative consequences on mothers and girls, especially on the health and psychological aspects, I will welcome and support the complete abandonment of FGM/C through strengthening of preventive measures or reducing practices that enforce it, like telling people that it is not a must to do FGM/C in the religion, that there are no religious obligations or laws that says it should be done and that it has negative effects on health.” Medical Doctor, Borama

“The community practices the Islamic religion and what they do is something that is good for the people. I want the Sunna one to be practiced. That’s my opinion as a practitioner and doctor. No, it does not have any problems. Every day it is practiced and there is no problem … It’s healthy and it is good for the body.” Medical Doctor, Hargeisa

##### Medicalization of FGM/C by health professionals

Participants reported that the medicalization of FGM/C was occurring in Somaliland, and was focused on the Sunna cut. Health professionals performing such work included nurses, midwives and medical doctors, with the cut occurring at health facilities or at individuals’ homes:

“Yes, there was change. Modern instruments are used in doing it nowadays. It is done by some health workers, such as nurses, who go to the homes of the mothers to do it for them.” Clinical Manager, Borama

“Mostly it is nurses and midwives who do it … They do the Sunna one only. It is mostly done in the nursing homes and MCH (maternal and child health), or the health centres and mostly doctors don’t do it. It is a light operation; anyone who is within the hospital can do it.” Nurse, Hargeisa

This medicalisation of the practice is also reported as more common in urban than rural areas. As one doctor observed:

“Those mothers who do it are mothers or midwives who have learnt the practice from other mothers. So, there are some who do it in the town who may be health workers, such as doctors, and they have something to stitch with and stop the bleeding but those in rural areas are mothers who have nothing in their hands. They have only thorns and razor blade. So, when they cut, there is nothing to stop the bleeding.” Medical Doctor, Hargeisa

#### Institutional level

At the institutional level, we explored capacity issues in terms of: the availability of health facilities to manage FGM/C complications; availability of FGM/C educational programs; availability of supplies and equipment; availability of clinical guidelines for managing FGM/C complications, and; the documentation of FGM/C statistics.

##### Availability of health facilities for managing FGM/C complications

Healthcare providers highlighted the lack of health facilities to manage FGM/C complications, particularly in rural areas where the practice is common.

“First, you need to know that healthcare is concentrated in urban areas and our people are more in the rural areas … The rural areas have few hospitals with no professionals. You can only find a midwife who has got little training, maybe six months training. Auxiliary nurses and midwives are mostly there in the better facilities. So, the main challenge is the facilities are not available everywhere and well-trained professionals are not there and if they are there, I can say they are few.” Medical Doctor, Borama

“In the rural areas, there are no well-trained health workers, but the women are assisted by midwives who do not have equipment and enough medicine to help girls with the complications.” Medical Doctor, Hargeisa

##### Availability of FGM/C educational programmes

A few healthcare providers were involved in awareness creation programmes aimed at educating community members on FGM/C complications within their facility and community. Community educational programmes were more common in urban than rural health facilities.

"The health system can prevent the practice of FGM by doing awareness creation. Now, it’s involved in the creation of awareness for the people by educating them about the problems of FGM/C.’ System Administrator 1, Borama

“The ability of the healthcare system is good here but in the rural areas it’s not. The system that is there in clinics is to do sensitisation for the people and educate them about impacts of FGM/C on the lives of people.” Medical Doctor, Hargeisa

##### Availability of supplies and equipment

The majority of respondents reported healthcare facilities lack the appropriate supplies and equipment to manage FGM/C complications successfully and in a timely manner. Supplies and equipment are expensive and most facilities are under-resourced, especially rural facilities.

“Most facilities don’t have life-saving equipment and medicines and those that have are expensive for the people to get.” Medical Doctor, Hargeisa

“There are inadequate supplies in health facilities, especially those in the rural areas.” Clinical Manager, Hargeisa

##### Availability of clinical guidelines for managing FGM/C complications

Effective treatment and management of FGM/C complications is partly informed by the availability of clinical guidelines. Respondents were unaware of any such guidelines that could assist them in the management of FGM/C complications. Consequently, interventions known to be effective in addressing such complications were not routinely available at facilities.

“I haven’t seen written guidelines or protocol. It is possible it could be there, but I haven’t heard nor have I seen a written protocol on treating the complications of FGM/C.” Medical Doctor, Borama

“There are no protocols and standard operating procedures for managing complications.” Clinical Manager, Borama

##### Documentation of FGM/C statistics

Healthcare professionals reported lack of documentation of FGM/C statistics. There were medical records of patients with complications likely to be FGM/C-related but there was no specific information captured to enable documentation of FGM/C cases in health facilities.

“There is none. There is no record. Most of our people talk about it but no documentation. There is nothing that they put into writing … The healthcare provider does not think he should record the statistics or do documentation.” Midwife, Hargeisa

“There is no statistics or records or data on FGM/C in the hospitals that are done.” Clinical Manager, Borama

#### Policy level

At the policy level, the study investigated: the availability of national policies for the management of FGM/C complications and against medicalisation of the practice, and; government financial investments and inter-sectoral linkages by the government for healthcare professionals to implement FGM/C treatment and management strategies.

##### National policies for the management of FGM/C complications and against its medicalization

Respondents were unaware of any national FGM/C management or anti-medicalization policy. A few reported the existence of a draft policy that had not yet been approved by parliament for implementation.

“There is no policy that I know for the management and care of the women, but we treat those who have complications of FGM/C.” Clinical Manager, Hargeisa

“No policy on FGM … The draft anti-medicalization policy is there but not passed in parliament.” System Administrator, Hargeisa

“I think it is there, there is a written draft government policy on FGM/C, but it has not been approved by parliament and no implementation is there.” Medical Doctor, Borama

##### Government financial investment and inter-sectoral linkages

Respondents felt that healthcare services were not prioritized by the Somaliland government. Consequently, resources—including for the capacity of healthcare workers and health facilities, especially in rural areas—to address FGM/C were limited.

“I think it’s about funds … there is one hospital in Borama, so it will be good to find it everywhere. Now it is only possible for people to come from far places to Borama [hospital, which is capable of handling fistulas]. It could have been better if every county/province had such a hospital and people who can educate them. Girls with FGM/C problems mostly don’t come to the health facilities, so it’s not given much attention.” System Administrator, Borama

Most facilities that could manage FGM/C complications were located in urban centers, despite the fact that most patients with FGM/C-related complications lived in rural areas where infibulation is commonly practiced. Respondents also reported poor inter-sectoral coordination and collaboration between health facilities run by non-governmental organizations and those run by the government.

“First, you need to know that healthcare is concentrated in urban areas and our people are more in the rural areas. The place where FGM/C is done mostly is the rural areas and small towns. The rural areas have few hospitals with no professionals … So, the main challenge is the facilities are not available everywhere and well-trained professionals are not there and if they are there, I can say they are few. There is also lack of coordination and collaboration between hospitals—government and organizations.” Medical Doctor, Borama

## Discussion

Healthcare workers play a critical role in the prevention, treatment and management of FGM/C complications. Essential prerequisites for them to play this role are their knowledge of those complications, their professional and personal attitudes and skills in handling them, the availability of health system resources to facilitate prevention and treatment, and a supportive environment. This study aimed to explore the knowledge, training and skills of healthcare workers and examined the current capacity of the healthcare system in Somaliland to prevent the medicalization of FGM/C and manage FGM/C-related complications generally. It found the system is confronted with multiple contextual challenges existing at the individual, institutional, and policy levels.

The study revealed that healthcare professionals in Somaliland were aware of the harmful physical, psychological and social consequences of FGM/C. These findings echo findings from a baseline study in the region that reported the majority of healthcare workers were well informed of FGM/C-related complications and health risks [17]. Nonetheless, healthcare providers managing girls and women with FGM/C face multiple challenges in their work, especially given the practice is not accorded the necessary governmental attention and resources. Most patients with FGM/C-related complications live in rural areas, where there are low-level, under-resourced facilities. Furthermore, providers lack the required training, skills and support to prevent FGM/C and manage its complications. Key Knowledge gaps remain for both the prevention of FGM/C and evidence-based care to optimize health outcomes for cut girls and women [52]. Healthcare providers in Somaliland should therefore receive comprehensive training to diagnose and treat FGM/C-related complications. There is need to develop and strengthen their capacity to manage all FGM/C complications effectively and prevent the medicalization of the practice within and outside their clinical settings. Special attention in this regard must be devoted to personnel in rural areas, where resources are minimal and where the most severe form of FGM/C (infibulation) is almost universally practiced.

Despite the availability of WHO guidelines on the management of FGM/C-related health complications, health facilities in Somaliland lack prevention and management guidelines. Clinical guidelines are critical in aiding health providers manage these complications [53, 54]. In the facilities in Borama and Hargeisa, such guidelines are unavailable for healthcare professionals to utilize, making routine prevention and management interventions problematic. Without the necessary guidelines to inform care, interventions aimed at preventing and managing FGM/C complications will be ineffective, especially in a republic with a comparatively high prevalence of infibulation and where the effective integration of FGM/C in existing health programmes is also lacking.

Clinical record keeping is an integral component in good professional practice and the delivery of quality healthcare [55]. At the individual level, documenting whether female patients have undergone FGM/C can potentially alert other healthcare providers to a woman’s FGM/C status and potential risks, thereby also avoiding unnecessary future intrusive genital examinations and improving the care experience [56]. Moreover, health information systems that link patient-level data on FGM/C, health facility data statistics, and population-level statistics on FGM/C is vital in the prevention and management of FGM/C-linked complications. Consistent with the findings of Johansen et al., who found that Somaliland has no designated medical code on FGM/C [57], healthcare professions in this study reported there was no registration and documentation system for FGM/C cases. The introduction of systematic integrated monitoring and evaluation data collection systems into those services that address the needs of girls and women with FGM/C is needed. An incorporated routine indicator will enable documentation of the precise nature and extent of FGM/C being undertaken and ensure the availability of accurate and up-to-date data on FGM/C in health facilities to inform investments, programmes and policies.

National policies on FGM/C signify governmental commitment to the prevention and provision of care for those at risk of FGM/C and those who have been subjected to FGM/C [57]. Our study revealed that Somaliland lacks a legislative framework and health policies that provide guidance on FGM/C care and against its medicalization; only draft policies existed that had neither been adopted nor approved. Lack of political commitment to approve these policies has been heightened by disagreement among national bodies calling for zero tolerance of FGM/C and religious leaders supporting the Sunna type of cut [58]. It is necessary to provide a coherent legislative framework on FGM/C prevention and management for the healthcare system to promote the protection of human rights against harmful practices and enhance the quality of health services available for girls and women living with FGM/C. However, it is clear such changes will have to occur in tandem with efforts to secure the support of religious leaders.

This study has several limitations: the transferability of the findings from Somaliland (North West) to other zones of Somalia (South Central and North East), with comparable FGM/C prevalence rates, is limited and merits further exploration to determine any comparability in findings. Second, the sample size of our exploratory study only included 20 participants. However, data collection through thematic saturation produced rich data that represented the viewpoints of health workers, clinical managers and system administrators at all levels of the healthcare system. Moreover, the study has identified the merit of additional future research that enumerates the major thematic areas highlighted by its findings.

## Conclusions

This study has addressed an important gap in the literature on FGM/C in Somaliland by providing an in-depth insight into the capacity of the healthcare system to manage FGM/C-related health complications and prevent the medicalization of the practice. In a republic where FGM/C is almost universally practiced, it is critical for the government to invest in FGM/C preventative and management strategies by incorporating the care of girls and women with FGM/C-related complications into all primary healthcare services through multi-sectoral collaboration and coordination. Moreover, there is need for more research to generate evidence on effective FGM/C prevention approaches, strategies to prevent the medicalization of the practice, and clinical management approaches and policy frameworks specific to the Somaliland context.

## Supplementary information

**Additional file 1.** Topic guide: FGM/C knowledge, attitudes, skills and training needs (facility staff).

## Data Availability

The research datasets generated and analyzed during this study are available from the corresponding author on reasonable request.

## Abbreviations

DFID: Department for International Development
FGM/C: Female Genital Mutilation/Cutting
TBA: Traditional Birth Attendant
UNICEF: United Nations Children’s Fund
WHO: World Health Organization
