# Supplementary material for: Exploring the capacity of the Somaliland healthcare system to manage female genital mutilation / cutting-related complications and prevent the medicalization of the practice: a cross-sectional study
Source: BMC Health Serv Res. 2020 Mar 12;20:200. doi: 10.1186/s12913-020-5049-2 (PMC7068950; doi:10.1186/s12913-020-5049-2)
Supplement: Supplementary file 1 — Additional file 1. Topic guide: FGM/C knowledge, attitudes, skills and training needs (facility staff). [file 12913_2020_5049_MOESM1_ESM.docx]

**Additional file 1: Topic guide: FGM/C knowledge, attitudes, skills and training needs (facility staff)**

1. In general terms, what is the level of your preparedness in terms of knowledge, attitudes, skills and training to manage clients undergone FGM/C?
2. More specifically, in terms of your knowledge of the practice, can you tell me some of the main reasons clients give for having the practice performed?
   1. What are the main factors and drivers?

PROBE FOR: ignorance of FGM/C practice and its consequences, social acceptance of FGM/C, ignorance of legislation and the legal status of FGM/C (does the law play any deterrent role?), actively supporting it (for various reasons), etc. Why do they continue to follow the practice in whatever form? Religion?

- 1. Does the decision come from them alone, or are there any other influences in your opinion? If the latter, what are they? PROBE FOR; Who is the most important influencer?
  2. Do you think FGM/C has changed from previous practices?

PROBE FOR: cutting at younger age, reduced severity of cut, medicalization of procedures etc? Reasons for the change?

1. Can you tell me some, if in your opinion there are any, of the health consequences of having the practice performed?
   1. PROBE FOR: Immediate; gynecological; obstetric; psychological; sexual and social consequences
   2. How easy or difficulty is it to manage the complications associated with FGM/C?

PROBE FOR; Reasons why they think it is easy? Reasons why they think it is difficult?

- 1. What are the challenges in managing the complications associated with FGM/C

PROBE FOR: Training deficits; hospital supplies; clients present late with advance complications; No skills; No functional referral system, lack of support from colleagues and supervisors; problem is not given the attention it deserves?

1. What is your opinion about the continuation or discontinuation of the practice?

PROBE FOR: Reasons why they think it should continue; Reasons why they think it should discontinue?

1. What do you think of the various strategies and programs to end the FGM/C?
2. PROBE FOR: Roles of Islamic leaders in the strategies; are they working/effective?
3. PROBE FOR: Roles of health care providers in the strategies; are they working/effective?
4. What do you think of those mothers / fathers who decide not to undergo FGM/C at all (neither traditional nor medicalised)?
   1. Why do you think that? Is it an opinion shared by many healthcare practitioners?
5. What is your opinion on the skills and training to handle clinical problems of women who have undergone FGM/C?

PROBE FOR: what skills they possess? What training they possess? Where they acquired them? In-service training? Preservice training? What the training entailed?

1. What training do you think you need to acquire the skills necessary to manage FGM/C requests?

PROBE FOR: training on complications of FGM/C? Addressing FGM/C complications?

PROBE FOR: what skills required to make them address FGM/C complications? De-infibulation? Counselling? Clitoral reconstructions? Others…

1. What is your opinion on the capacity of health care system to respond to women undergone the cut?

PROBE FOR: adequate trained health care personnel? Adequate supplies? Availability of protocols and standard operating procedures? Availability of cascaded policy on FGM/C care? Supportive supervision? Anchoring of FGM/C on existing health programs?

1. What is your opinion on the capacity of health care system to prevent women and girls from undergoing the cutting?

PROBE FOR: availability of government policy on FGM/C; availability of demedicalization policy; availability of anti-FGM/C prohibition law? Integration of FGM/C in the existing health programs? Availability of records/statistics of health talks on FGM/C? Availability of statistics on FGM/C and linkage with maternal/infant health?

1. What systems are in place for giving feedback on the cases, management and outcomes of FGM/C case management in this facility?
2. What systems are in place which incorporates FGM/C statistics to the facility health statistics and the general county statistics in terms of FGM/C practice? Explain?
3. What referral system is there for FGM/C?
4. What supervisory system is available for FGM/C?
5. What policy are there for management and care of women and girls with FGM/C
6. Is there anything we didn’t talk about which you think it would be useful to know?
